# Creating a healthier economy: A rapid evidence review of inequalities in flexible working arrangements in the UK

**DOI:** 10.1016/j.puhip.2025.100649

**Published:** 2025-08-20

**Authors:** A. Barnes, C. Cartwright, K. Kennedy, A. Formby

**Affiliations:** a Healthy Livelihoods - Public Health and Society, Health Sciences, University of York, UK; b Research Programme Director, Bradford Institute for Health Research, Bradford Teaching Hospitals NHS Foundation Trust, Duckworth Lane, Bradford, UK

**Keywords:** Healthy economy, Flexible working, Power and control, Public health, Inequality

## Abstract

**Objectives:**

Flexible working – the control people have over work scheduling to meet wellbeing needs – is one way to help create a healthier economy. We sought to identify and summarise evidence about inequalities in access to flexible working arrangements in the UK and implications for health and wellbeing to inform policy development.

**Study design:**

Rapid evidence review.

**Methods:**

A rapid review of peer-reviewed articles and reports from trusted sources was completed on inequalities in access to UK flexible working arrangements to inform regional and local policy development. Published articles were identified through database searches (OVID-Embase, Scopus, Cochrane, Assia-Proquest) in September–October 2024. Data was extracted directly into a table and findings synthesised narratively by theme.

**Results:**

Evidence identified was limited in detail, but consistent in reporting inequalities in access to flexible working by occupational status, with some evidence of inequalities by gender, disability, geography and ethnicity; with implications for health promotion. There was limited detail on specific health outcomes or pathways to impacts, though the significance of flexible working for women's well-being and Disabled people was highlighted. Included evidence noted systemic issues in the UK economy (e.g. occupational hierarchies, gendered norms about caring, racism, disability discrimination, ‘ideal worker’ culture that values overwork, flexibility stigma) that contribute to flexible working inequalities.

**Conclusions:**

Further research and multi-level policy action is needed to address flexible working inequalities to promote health. Research could usefully focus on intersectional inequality, including systemic societal processes (i.e. stigma, racism, discrimination) shaping flexible working in practice. Policy action could include: evaluating the implementation of existing flexible working legislation in relation to inequality; national-regional-local action to support inclusive business models in which the bargaining positions of employees around flexibility are more equalised (e.g. cooperatives); evaluation and strengthening of Fair Work Charters; and funding and showcasing of flexible working pilots focused on addressing unequal flexible working access.

## 1 What this study adds

1. First rapid review to draw together evidence to understand inequalities in access to flexible working in the UK, including implications for health and wellbeing
2. Identifies a limited evidence base (largely quantitative studies) but consistent reporting of inequalities by occupational status, with some evidence of inequalities relating to gender, disability, geography and ethnicity, which has implications for health promotion
3. Inequalities identified reflect systemic issues within the UK economy (i.e. occupational hierarchies, gendered norms about care, racism, disability discrimination, ‘ideal worker’ culture that values overwork, flexibility stigma) - future research could usefully focus on the intersection of these issues with flexible working and understanding pathways to impacts, using qualitative, mixed-methods and theory-informed approaches

## 2 Implications for policy and practice

1. Inclusive access to flexible working needs to remain a central feature of work and health-related policy discussion about creating a healthier economy
2. Regional and local policy action could include: evaluating the implementation of voluntary Fair Work Charters and strengthening Charter provisions on flexible working (e.g. asking for commitments to monitor uptake and report on inequalities); business support teams providing advice on flexible working and exploring options to grow enterprises in which the bargaining positions of employees around flexibility are more equalised (e.g. cooperatives); and funding and showcasing pilots that address unequal access
3. National-level policy actions to help address inequality could include: evaluation of the implementation of existing flexible working legislation in relation to inequality; further legislation to ensure workers have control over the predictability of working hours; action to support business models in which the bargaining positions of employees around flexibility are more equalised (e.g. cooperatives); and investment in flexible working pilots to address inequality in different sectors of the economy

## 3 Introduction

Creating a healthier economy – the way we produce and provide for one another – is a key population health challenge [1]. Our current economic system prioritises economic growth and private profit, sometimes at the expense of health and wellbeing: for example, by damaging the environmental systems upon which our wellbeing depends, and concentrating (versus distributing) income, social status and wealth, thus undermining people's agency and control over the decisions that impact their lives and health [2,3]. Various multi-sectoral actions have been suggested to move towards a more health-promoting economy, including: shifting the economy's purpose away from Gross Domestic Product (GDP) towards wellbeing, building opportunities for more diverse social groups to participate in economic decision-making, reforming social security systems to ensure a basic standard of living for everyone, and growing ‘healthier enterprises’ that more fairly *pre*distribute people's power and control, income and/or time, including through living wages and flexible working [4].

**Fig. 1:**
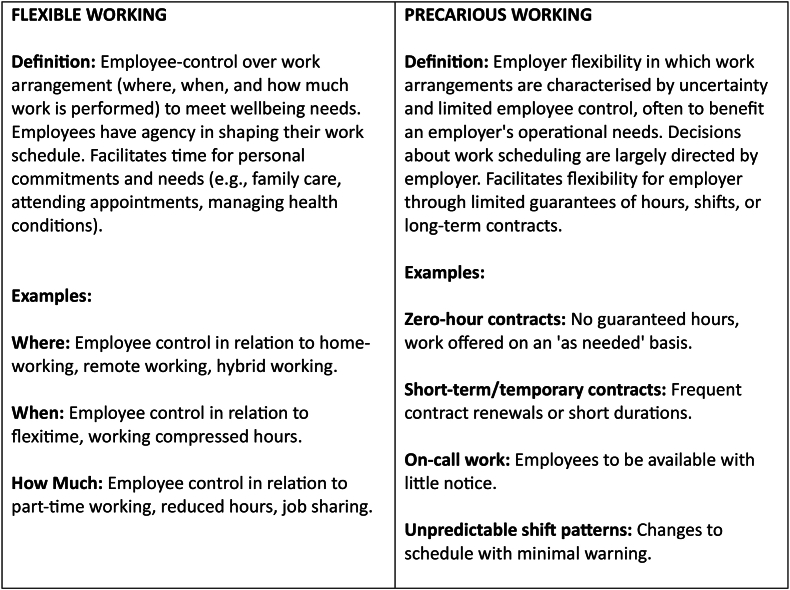
Definition of flexible working as compared with precarious working (Source: author generated).

Flexible working is about the control people have over work scheduling to meet wellbeing needs, including where (e.g. home-, remote- and hybrid-working), when (e.g. flexitime) and how much work (e.g. part-time work) is carried out [5]. Flexible working contrasts with precarious working as form of employer flexibility, in which work arrangements are characterised by uncertainty and limited employee control over work scheduling (see Fig. 1). There is increasing evidence that the level of control we have in our lives, including workplaces, is important for health, with lack of control a driver of public health inequalities [[6], [7], [8]]. In some workplaces, there is a graded relationship between people's status and the control they have, with lower control linked to higher stress, increased rates of sickness absence, poorer mental health and higher risks of cardiovascular disease [[8], [9], [10]]. Conversely, when employees are enabled to have control over work scheduling this can promote health [11]; for example, by allowing people to socialise with and/or care for family and friends, attend medical appointments, and manage disabilities, including long-term health conditions. For employers, flexible working can have reciprocal benefits, with control over work scheduling leading to improvements in employee motivation, staff retention, and productivity [12,13].

In the UK, in April 2024, employees gained the right to request flexible working in relation to their hours, working times or work location from the first day of employment [14,15]. Employers are required to deal with requests in a ‘reasonable manner’, but relatively little is known about inequalities in how requests are dealt with for different employees, how flexible working is supported in different sectors of the UK economy, or the effect on inequalities in health and wellbeing.

Since the start of the COVID-19 pandemic, an increasing number of international reviews have focused on exploring the effects of home, hybrid or remote working on health [16,17], but their starting points have not always been flexible working defined as a form of employee control (e.g. they have considered home working arrangements that are employer-driven or ‘required’ due to the pandemic as opposed to flexible working as form of worker control), and few have explicitly considered inequalities in access (e.g. by gender, ethnicity, disability, neurodiversity), despite emerging evidence that flexible working may not always be implemented inclusively [18]. In the UK, for example, the British Chamber of Commerce reported in 2023 that three quarters of UK businesses offered flexible working, but with significant differences across sectors - flexible working was found to be less likely in manufacturing, retail and hospitality [19]. At the time of writing, no review had focused specifically on inequalities in access to flexible working arrangements in the UK by population group. Yet understanding more about this is particularly important in a UK context given the high numbers of working-age people (over 2.8 million in October 2024) reporting long-term health conditions that affect their economic participation, and government interest in moving towards an 80 % employment rate [20]. Access to control over work scheduling is a potentially important way of enabling this group to return to paid employment in a way that works for them and their wellbeing.

It is in this context that local and regional decision-makers are developing policies to build a fairer economy, in which everyone has access to employment security and flexible working [21]. To this end, initiatives such as Fair Work Charters have been introduced in some areas in England asking participating employers to confirm they have taken steps towards implementing flexible working (amongst other areas of workplace action) [21]. However, decision-makers have limited access to UK evidence about inequalities in flexible working which could inform this and other types of policy action on flexible working. Given this gap, a rapid review was completed to particularly inform local and regional policy development, but also with relevance to national action on flexible working. The aim was to rapidly summarise evidence on inequalities in flexible working arrangements in the UK, including any insights about health and wellbeing.

## 4 Methods

A rapid evidence review was completed within limited time (two months) and budget to provide insights for local and regional policymaking. Regional policy makers indicated the need for the work and were consulted to agree the scope (see Acknowledgements section). Rapid reviews adjust systematic review methods to balance academic rigour with expediting evidence into practice when needed [22]. Standard rapid review methods do not exist because of the need to adapt methods to meet practice needs. Relevant rapid review guidance was therefore used to complete the review [22,23].

### 4.1 Searches

Four electronic databases were searched in October 2024 (OVID-Embase, Scopus, Cochrane, Assia-Proquest) using terms relating to flexible working (Supplementary File 1). Advice was sought from an information specialist before completing the searches. Database searching was supplemented by hand-searching websites of key trusted sources identified as relevant through discussion with West Yorkshire policymakers (see Supplementary File 1). Key author searching (Chung) and reference checking of included sources was also completed.

### 4.2 Inclusion criteria

The rapid review included: peer-reviewed journal articles of any type (except systematic reviews unless the review clearly focused on the UK) or reports from key trusted sources, which had insights about inequalities in flexible working arrangements in the UK, including relating to access or health and wellbeing; written in English; and published since 2014.

### 4.3 Screening and selection of reviews

One reviewer screened titles and abstracts against inclusion criteria. There was insufficient resource for second checking, which is a common limitation of rapid reviews. Two-stage screening was used, with initial identification of possible in scope sources for full document review. Evidence excluded at full document review was recorded, with reasons, in a table (Supplementary File 2).

### 4.4 Data extraction and synthesis

Key information in included evidence was extracted directly into a table to rapidly complete the review to meet regional policymaker needs. Information included: authorship, publication date, title, source type, methods, the forms of flexible working considered (e.g. control over hours/location), the forms of discrimination or disadvantage mentioned in relation to flexible working (e.g. relating to gender, occupational status, disability) and key points made. Insights from all included sources were summarised and synthesised narratively in relation to the different forms of inequality mentioned.

### 4.5 Evidence quality

We used appraisal checklists to consider the quality of included literature (Supplementary File 3). Only a small proportion of reported data was relevant in many included journal articles, with a lack of detailed published evidence overall focusing *specifically* on inequalities in access to flexible working and with limited data about health or health inequality implications. We also included unpublished reports from trusted sources, which were often unclear about underpinning methods or evidence. We include general reflection on evidence quality in the discussion as it also considers limitations.

## 5 Results

**Fig. 2:**
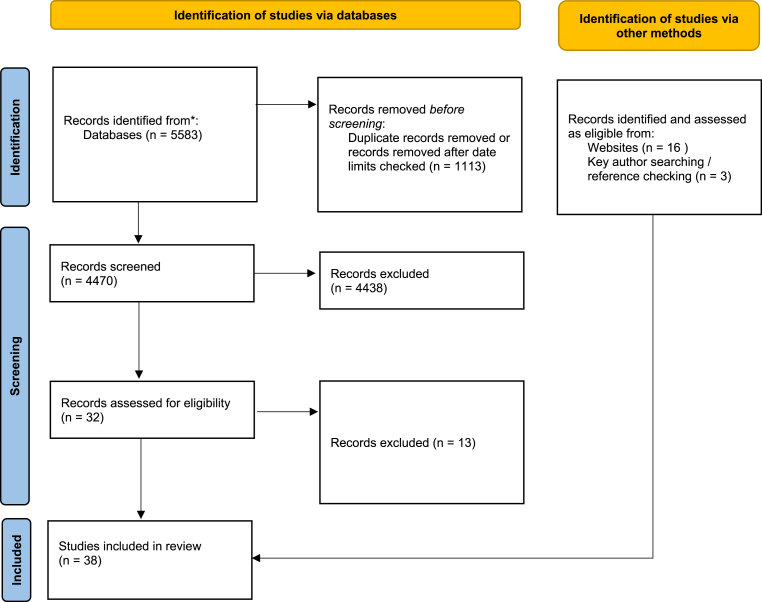
PRISMA diagram.

Source: Page MJ et al. BMJ 2021; 372:n71.https://doi.org/10.1136/bmj.n71.This work is licensed under CC BY 4.0. To view a copy of this license, visit https://creativecommons.org/licenses/by/4.0/

A total of 38 sources were included: 22 peer-reviewed articles and 16 reports (Fig. 2). Of these, 22 were quantitative studies, 5 mixed methods, 3 qualitative, and 4 literature reviews (with unclear methods). Seventeen papers mentioned multiple different types of inequality (though most did not specifically seek to explore inequality in flexible working), 18 papers addressed issues of gender inequality in some way, 10 addressed disability, 9 inequality and occupational status, 3 geographical inequality, and 3 racialised or ethnicity-related inequality and flexible working (Supplementary File 2 summarises characteristics of all included sources).

### 5.1 Synthesis of findings

Although detailed evidence was limited overall, there was consistent reporting of inequalities in access to flexible working arrangements, with implications for health promotion. These were categorizable by five themes as below.

#### 5.1.1 Inequalities by occupational status

Included evidence consistently reported inequalities in access to flexible working arrangements by occupational status: people in roles lower in a skills-status hierarchy, lower paid, and with less authority and bargaining power, were reported to be less likely to have access to flexible working, despite potentially being those most need of it due to lack of access to other resources (e.g. finances) to address conflicting work-life demands [13,[24], [25], [26], [27], [28], [29]]. For example, those in lower-paid roles (e.g. clerical, administrative assistants) were reported to have less control over work location (e.g. home, hybrid working) where roles permitted it (i.e. no business need to be physically present on site) [13,26,28,30]. Lower status employees were also reported to have lower levels of control over working times (flexitime) and informal time-off work for personal reasons [24,25,27]. In one European-wide study, one upwards move in the skill hierarchy was found to increase the likelihood of being able to access schedule control by approximately 1.5 times [25].

In relation to home and hybrid working specifically, one report noted that existing UK patterns of digital exclusion (i.e. poor access to digital infrastructure such as a computer, software, internet) could contribute to inequalities in access to home and hybrid remote working [13]. People on low incomes or with migrant backgrounds, lower educational attainment, Disabled people, and/or from rural communities, are more likely to be affected by digital exclusion and thus potentially less likely be able to work remotely where job roles allow it [13]. It was reported that rises in UK remote, hybrid and homeworking during and after the COVID-19 pandemic were mostly amongst managerial, technical, and professional occupational groups, though with some increase in access among groups where homeworking had previously been rare (e.g. call centre workers, administrative staff, clerical assistants, sales clerks – groups not usually regarded as privileged in the labour market) [13]. Yet it was also indicated that increases in hybrid or homeworking in these groups were less likely to be voluntary (i.e. did not reflect flexible working as control) and were potentially linked to weakening employee autonomy, remote monitoring and surveillance [13]. This kind of employer-driven flexibility, which undermines autonomy and control, could have negative implications for employee health and wellbeing.

#### 5.1.2 Geographical and sectoral inequalities

Three reports from trusted sources highlighted UK geographical inequalities in access to flexible working [13,31,32], with employees in Yorkshire and Humber, for example, having some of the lowest access to control over when and where to work, including informally taking time off during work to deal with personal matters at short notice [31]. This kind of schedule control can be particularly important for parents, unpaid carers and people with disabilities, including long-term health conditions [32,33] and there may therefore be wellbeing implications of such inequality, though this was not considered in detail in included sources. A complex mix of factors (e.g. historical trends in the geographic location of particular sectors and higher status professions, combined with sectoral inequalities in dealing with flexible working requests, see below) likely contribute to geographical inequalities in access to flexible working [27,29,31].

It is usually possible for all employers to enable employees with some control over work scheduling to meet wellbeing needs (e.g. control over number of hours worked, shift patterns, rostering, working hours reliability). However, included sources reported difficulties for employees in some sectors in securing support for flexible working requests [29,34]. Issues were reported, for example, in accessing reduced working hours in the police [34] and control over shift rostering within the health service [29]. Included reports shared examples of action taken by UK employers in retail, transportation (HGV) and health service to improve access to flexible working for frontline and site-based employees (e.g. piloting new ways of job-sharing, team-based rota planning), with positive impacts on staff turnover, perceptions of fairness and worker productivity [13,29]. Limited information was provided in included sources on wider implications for health and wellbeing.

#### 5.1.3 Gendered inequalities

Included evidence reported that men and women may use and/or experience flexible working in distinctive ways, with important implications for inequalities at home and in workplaces, and for health and wellbeing [13]. Having some control over work scheduling is particularly important for women's wellbeing, given they often have more family demands than men due to gendered societal norms about women providing care [13,24,[35], [36], [37], [38]]. Included sources reported that UK women can experience wellbeing benefits (including improvements in subjective wellbeing (life, leisure satisfaction), lower anxiety, depression, chronic stress), from access to control over when, how much and how reliable work is (e.g. fixed working hours/days) – enabling better management of paid work and unpaid caring responsibilities and to stay in or get back into paid work (for example, after childbirth) [13,24,[35], [36], [37]].

One study reported that control over working hours (e.g. working the same number of hours across fewer days) was particularly important for the mental wellbeing (e.g. lower levels of anxiety and depression, improved job satisfaction) of single working mothers, enabling the meeting of caring responsibilities without financial consequence [37]. Another study indicated that control over working hours may be particularly important for women during menopause, with differential access potentially translating into inequalities in how menopausal symptoms are managed and feelings of overwhelm during this important transitional period in women's lives [39].

There was some evidence to suggest that there may be gendered inequalities in access to flexible working. Amongst those working in professional occupations, one study reported that women had less access to informal flexibility than men [27] and a report noted that UK men in professional job roles may be more likely to remote work than UK women (though women are more likely to work from home) despite evidence suggesting that women are more likely to have professional job roles that can be undertaken remotely [13]. Reduced hours flexibility (e.g. part-time working) is particularly used by UK women who are responsible for caring for children under the age of 15,^37,40,41^ but included evidence suggests that this can intensify women's disproportionate share of household tasks (e.g. cooking, cleaning, washing) and informal care, with negative impacts on women's income and career [13,40] which is an important determinant of long-term health. Here, ‘flexibility stigma’ (negative perceptions or valuing of those who work flexibly for family purposes) may contribute to negative impacts on career progression and promotion, with mothers more likely to say they had experienced negative career outcomes due to working flexibly [36,41]. Flexibility stigma was reported to be prevalent in the UK before the COVID-19 pandemic [41]. Yet men who access flexible working arrangements face flexibility stigma and potentially also ‘femininity stigma’ given how accessing flexible working for care purposes can lead to men being perceived as deviating away from ‘ideal worker’ norms that value overwork and normalised views about who should provide care [41] – this may also mean that some men may not feel comfortable in submitting flexible working requests.

There is some evidence to suggest that home working can reinforce traditional gender roles and gender pay gaps during parenthood: while it may help women to better manage caring responsibilities, boundaries between work and family life can blur leading to higher levels of work-family conflict, which can undermine family wellbeing [13]. One report noted that the rise in UK home working, especially by fathers, during the COVID-19 pandemic could contribute towards more equitable population-level patterns of domestic labour and care, helping remove stigma against flexible working - though this was reported to be unlikely to be sufficient to combat inequalities [13]. In contrast, for men and women *without* children or other caring responsibilities, remote and home working may enable work intensification (e.g. longer working hours, breaks, being constantly available) [35,42]. Such intensification can lead to stress and fatigue, but also contributes to income premiums (i.e. supporting pay and progression), given how this kind of ‘unpaid overtime’ is a strong determinant of promotion in the UK [35].

#### 5.1.4 Flexible working inequalities, racism and ethnicity

Only two included sources mentioned inequalities in flexible working arrangements for people of minoritised ethnicities. Both sources noted how racialised occupational segregation in the UK labour market meant that employees of minoritised ethnicities were over-represented in lower paid, frontline, and often physically-demanding roles in which working from home may not be possible, and/or in lower status occupations that tend to afford fewer opportunities for autonomy and control over work-time scheduling arrangements [18,29]. A recent unpublished report indicated that men who identify as Black (African/Caribbean/Black British), Chinese/other Asian or Pakistani/Bangladeshi were amongst the least likely to state home as their main place of work, with a similar pattern found for women and parents in minoritised ethnic groups and with this patterning more evident since the COVID-19 pandemic [18]. The same report indicated that workplace racism (e.g. discriminatory perceptions about work ethic due to workplace racism) combined with flexibility stigma risked some minoritised employees feeling less able to ask for flexibility [18]. Yet home or hybrid working might provide employees of minoritised ethnicities with some respite from negative wellbeing effects of workplace racism and discrimination and/or from the mental stress of negotiating workplace cultures that do not value diversity (e.g. of dress, appearance) [18].

#### 5.1.5 Inequalities relating to disability

Flexible working can be an important means for working age Disabled people, including people living with long-term health conditions, to manage their wellbeing needs, enabling them to stay in or return to work, preventing isolation and reliance on social security/welfare benefits [13]. However, included sources reported inequalities in Disabled people's access to flexible working [32,[43], [44], [45], [46]]. It was reported, for example, that Disabled UK employees were less likely to work from home than non-disabled employees, given exclusions experienced from organisations and higher status managerial roles in which home working tends to be more available [45]. Some Disabled employees with access to home working experienced digital exclusion, including not having access to essential work equipment and/or using their own money to purchase it [43,47]. Other difficulties reported included negative attitudes from line managers about flexible working and being left out or isolated when working at home [43]. It was noted that home working can have important wellbeing benefits for Disabled people and people who are not disabled (e.g. job-related mental health, feeling more productive, job satisfaction, work-life balance, management of health conditions, reduced fatigue and tiredness) [43,45,47] but that there is no evidence that homeworking is associated with smaller disability gaps in these outcomes or that it could reduce disability disadvantage within the labour market [45].

Difficulties in accessing return-to-work flexibility (e.g. re-entering work on a reduced hours-flexible basis) were also reported for UK employees who had been unable to work due to poor health [13]. It was noted that lack of control over work scheduling was a key driver of the UK disability employment gap [43] and that Disabled employees were more likely to have changed jobs or career due to limited flexibility [48]. It was noted that well-trained line managers, and senior management and Human Resources support, can support inclusive access to flexible working [49].

Some Disabled people, those with caring responsibilities carers and/or people in minoritised ethnicities work in precarious, insecure jobs (e.g. on zero-hours contracts, in self-employment), partly due to difficulties in finding securely employed roles which allow for control over work schedules [33,44,50,51]. However, insecure jobs come with a consequent loss of important employment protections (e.g. sick pay, redundancy rights), and social security benefits (to top up low wages) are often negatively impacted by irregular income from zero-hour contracts [52].

## 6 Discussion

Flexible working – enabling employees to exert control over work scheduling – is one way of helping to grow a more health-promoting economy and address some forms of in-work inequality. While flexible working may be inclusively offered by many UK employers, our findings suggest that inequalities in access to flexible working arrangements exist, with a clear (though not extensive) body of evidence, largely quantitative studies, highlighting that lower status, lower paid workers are less likely to have this kind of control. A smaller body of included evidence suggests that Disabled employees and employees in minoritised ethnicities are at risk of unequal access to flexible working, and that there are gendered, geographical and sectoral inequalities in access to flexible working in the UK. These findings are consistent with wider international evidence that highlights inequities in the implementation of policies relating to employee flexibility [53,54].

Although the rapid review also sought to identify insights about how inequalities in access to flexible working arrangements relate to health and health inequalities, there was limited detail in included evidence on specific health outcomes or pathways to health impacts, though some evidence highlighting the particular significance of flexible working for women's wellbeing. A lack of research on direct health and wellbeing implications of flexible working has been identified in other international reviews and remains an area for future research [11]. Wider international review evidence on flexible working and employee health finds that flexible working is associated with better physical health, reduced issues of pain and fatigue, and reduced absenteeism [55]. International reviews also highlight, as our study has found, the broader importance of control over work scheduling for health promotion: for example, via pathways linked to having time to socialise with and/or provide care for family and friends without financial consequence, access health/care attend medical appointments, and/or manage long-term health conditions, in a way that works for them [55]. Given UK policy interest in supporting the high number of working-age people with long-term health conditions who are not in work into employment [[20], [56]], it is important that pathways to health are better understood and that inclusive access to flexible working remains a central feature of work and health-related policy debates.

On connections with women's wellbeing, included evidence was clear that gendered norms about women providing care mean it is particularly important for women to have access to control over work scheduling, with this particularly important at key transition points in women's lives (e.g. after the birth of a child, becoming a single parent, during menopause). In this way, flexible working is central to women's financial security, which is as an important determinant of health [57]. Yet our review findings also suggest that impacts of flexible working on women's wellbeing are complicated due to gendered norms that shape the division of labour within the home [38], and also mean that that some men find it difficult to access flexible working, with implications for families.

While there is always the risk in a rapid review of this kind that pertinent sources have not been included (because the aim was not to cover everything but to inform policy), our findings suggest that the current evidence base is dominated by quantitative research: something noted in other related international reviews [17]. More UK-focused research, using longitudinal, qualitative and mixed methods approaches to give multiple perspectives [58] and focusing on issues of gender, age, disability and ethnicity are clearly needed, and which are grounded in more robust theory of how access to flexible working can shape health and health inequalities - if we are to more fully understand and address the inequalities in flexible working identified and promote health. No evidence was identified that considered how intersectional forms of disadvantage might shape inequalities in access to flexible working. Future research could usefully take an intersectional lens, exploring how multiple forms of inequality might act together to influence how flexible working is accessed and experienced. Within this, we suggest a need to explore dominant ‘ideal worker’ norms in the UK that value overwork and social processes of stigmatisation (i.e. negative valuing of those who request or access flexible working), given findings about the adverse impacts of flexibility and femininity stigma on access to control over work scheduling, career trajectories and income, and thus health promotion.

Overall, we argue that inequalities identified in this review reflect systemic issues within the UK economy (i.e occupational hierarchies, racism and discrimination, ‘ideal worker’ culture that values overwork, flexibility stigma). Given this, a range of multi-level reforms are needed to increase inclusive access to flexible working, whilst ensuring that employers can access the talent they need to grow a healthier economy [20]. Nationally, evaluation of existing flexible working legislation in relation to inequality; further legislation to make fair work mandatory, including by ensuring that workers have control over the predictability of working hours (e.g. in the forthcoming Employment Rights Bill 2024-25) [33,59]; action to support inclusive businesses (e.g. cooperatives, employee-owned firms) in which bargaining positions of employees around flexibility tend to be more equalised [3], and investment in flexible working pilots to address inequality in different sectors of the economy could all be useful. Learning from pilots could also be mobilised nationally, regionally and locally to showcase good practice, building awareness about the possibility of enabling employees to have control over work scheduling whilst generating reciprocal benefits for employers (e.g. staff retention, productivity) [12,13].

In a context of further UK policy devolution, local and regional policymakers could appraise local and regional inequalities in access to flexible working, and consider a range of other policy initiatives to address identified issues, including, for example: monitoring and evaluating the implementation of Fair Work Charters and strengthening the provisions on flexible working (i.e. asking for employer commitments to monitor uptake and report on inequalities); investing in, supporting and ensuring that local and regional procurement processes support the growth of more inclusive businesses and improve the quality of work available, by building this action into a coherent local wealth building approach [60]; and ensuring that business support teams provide advice to managers and HR teams to normalise conversations about flexible working (as one step towards addressing flexibility stigma) and ensure organisational policies make clear that requesting flexible working is a right for everyone [61]. Yet it is clear that addressing issues of flexibility stigma and ensuring inclusive access to flexible working will also require wider policy and workplace actions to address the UK's ‘long hours’ working culture, normalise the involvement of men in providing care, and action to ensure that all employees feel safe, included and a sense of belonging in all working environments and can thrive within the economy.

## Author contributions

Barnes: Conceptualisation, Methodology, Investigation, Formal analysis, Data curation, Interpretation, Writing – Original Draft, Supervision, Approval of submitted version. Cartwright: Conceptualisation, Methodology, Resources, Interpretation, Writing – Review & Editing, Supervision, Approval of submitted version.

Kennedy: Interpretation, Writing – Review & Editing, Approval of submitted version.

Formby: Interpretation, Writing – Review & Editing, Approval of submitted version.

## Ethical approval

Ethical approval was not needed as the rapid review did not involve human participants.

## Funding

Cartwright and Barnes have received funding from the NIHR Yorkshire and Humber Applied Research Collaboration [reference NIHR200166]). Barnes and Kennedy have received funding from the UK Prevention Research Partnership Collaboration (MRC) - ActEarly [reference MR/S037527/1]. Barnes, Formby and Kennedy have received Research England Policy Support Funds distributed via The York Policy Engine at the University of York. The views expressed in this publication are those of the author(s) and not necessarily those of the National Institute for Health and Care Research, Department of Health and Social Care, UK Prevention Research Partnership/MRC, Research England, West Yorkshire Combined Authority, West Yorkshire Health and Care Partnership, Y-PERN or Yorkshire Universities.

## Declaration of competing interest

The authors declare that they have no known competing financial interests or personal relationships that could have appeared to influence the work reported in this paper.
