# Supplementary material for: Creating a healthier economy: A rapid evidence review of inequalities in flexible working arrangements in the UK
Source: Public Health Pract (Oxf). 2025 Aug 20;10:100649. doi: 10.1016/j.puhip.2025.100649 (PMC12446758; doi:10.1016/j.puhip.2025.100649)
Supplement: Multimedia component 1 [file mmc1.docx]

**Supplementary File 1 – Further detail on the rapid review search process**

**Example terms used in database searches**

The rapid review on inequalities was part of an associated rapid review carried out at the same time, exploring what is known about the connections between flexible working, health-economy-environment outcomes and inequalities. In order to meet the needs of practice, only one database search process was completed for both reviews, using a broad set of search terms that covered concepts relating to the economy and environment, as well as inequality. In practice, it meant a more comprehensive search and sift process than if concepts only relating to flexible working and inequality had been used.

| Search term domains | Scopus |
| --- | --- |
| Flexible working | TITLE-ABS-KEY  "flexible work*" OR flexitime OR flextime OR flexplace OR "remote work*" OR "schedule control*" OR telework OR telelcommut* OR "home work*" OR "work* from home" OR "hybrid work" OR "compressed hours" OR "flexi hours" OR "flexi-hours"  AND |
| Health | "mental health" OR "physical health" OR wellbeing OR "well-being" OR "quality of life" OR "informal care*" OR "unpaid care*" OR "control at work"  OR |
| Economy | productiv* OR "sickness absence" OR "staff retention"  OR |
| Inequality | Inequal* or Inequit*  OR |
| Environment | "climate change" OR "pollution" OR "carbon emission*" OR "congestion" |
| Language limit | English language |
| Other limits | From 2010 |

**List of key trusted sources whose websites were hand-searched to identify reports to include in the rapid review**

- Chartered Institute of Personnel and Development
- What Works Local Economic Growth
- Office for Health Improvement and Disparities
- ReWAGE expert group
- Timewise
- UK-based Trade Unions (e.g. TUC)
- Work Foundation
