# Supplementary material for: Creating a healthier economy: A rapid evidence review of inequalities in flexible working arrangements in the UK
Source: Public Health Pract (Oxf). 2025 Aug 20;10:100649. doi: 10.1016/j.puhip.2025.100649 (PMC12446758; doi:10.1016/j.puhip.2025.100649)
Supplement: Multimedia component 3 [file mmc3.docx]

**Supplementary File 3 - Quality appraisal**

For critical appraisal, we used checklists from the Critical Appraisal Skills Programme (Critical Appraisal Skills Programme: <https://casp-uk.net/casp-tools-checklists/>) relevant to each empirical study design, and the AACODS checklist for grey literature, to consider the quality of identified literature.

**Grey literature - AACODS checklist [modified]**

**0-5 rating 0=low/poor 5= high/good**

| **First author & date** | **Authority** | **Accuracy** | **Coverage** | **Objectivity** | **Date** | **Significance**  **(to specific research area that is a focus of the review)** | **TOTAL** |
| --- | --- | --- | --- | --- | --- | --- | --- |
| Allen et al, 2024 | 4 | 3 | 4 | 4 | 3 | 4 | 22 |
| Atay et al, 2023 | 5 | 4 | 4 | 4 | 5 | 4 | 26 |
| Atay et al 2024 | 5 | 2 | 2 | 3 | 3 | 4 | 19 |
| Chung et al., 2024 | 5 | 4 | 4 | 4 | 5 | 5 | 27 |
| CIPD, 2019 | 4 | 2 | 2 | 3 | 3 | 4 | 18 |
| CIPD, 2020 | 4 | 2 | 2 | 3 | 3 | 4 | 18 |
| CIPD, 2021 | 4 | 2 | 2 | 3 | 2 | 4 | 17 |
| CIPD, 2020 | 4 | 2 | 2 | 3 | 3 | 4 | 18 |
| Florisson, 2024 | 5 | 4 | 4 | 4 | 5 | 4 | 26 |
| Gable and Florisson, 2023 | 5 | 4 | 4 | 4 | 5 | 4 | 26 |
| Martin et al 2024 | 5 | 4 | 4 | 4 | 5 | 4 | 26 |
| ReWaGE, 2023 | 5 | 3 | 3 | 4 | 4 | 5 | 24 |
| Taylor et al., 2022 | 5 | 3 | 3 | 4 | 4 | 5 | 24 |
| Timewise, 2023 | 5 | 3 | 3 | 4 | 4 | 5 | 24 |
| TUC, 2021 | 4 | 2 | 2 | 3 | 4 | 4 | 19 |

**AUTHORITY**

**Individual author:**

• Associated with a reputable organisation?

• Professional qualifications or considerable experience?

• Produced/published other work (grey/black) in the field?

• Recognised expert, identified in other sources?

• Cited by others? (use Google Scholar as a quick check)

• Higher degree student under “expert” supervision?

**Organisation or group**:

• Is the organisation reputable? (e.g. W.H.O)

• Is the organisation an authority in the field?

**In all cases:**

• Does the item have a detailed reference list or bibliography?

**ACCURACY**

• Does the item have a clearly stated aim or brief?

• Is so, is this met?

• Does it have a stated methodology?

• If so, is it adhered to?

• Has it been peer-reviewed?

• Has it been edited by a reputable authority?

• Supported by authoritative, documented references or credible sources?

• Is it representative of work in the field?

• If No, is it a valid counterbalance?

• Is any data collection explicit and appropriate for the research?

• If item is secondary material (e.g. a policy brief of a technical report) refer to the original. Is it an accurate, unbiased interpretation or analysis?

**COVERAGE**

All items have parameters which define their content coverage. These limits might mean that a work refers to a particular population group, or that it excluded certain types of publication. A report could be designed to answer a particular question, or be based on statistics from a particular survey.

• Are any limits clearly stated?

**OBJECTIVITY**

It is important to identify bias, particularly if it is unstated or unacknowledged.

• Opinion, expert or otherwise, is still opinion: is the author’s standpoint clear?

• Does the work seem to be balanced in presentation?

**DATE**

For the item to inform your research, it needs to have a date that confirms relevance

• Does the item have a clearly stated date related to content? No easily discernible date is a strong concern.

• If no date is given, but can be closely ascertained, is there a valid reason for its absence?

• Check the bibliography: have key contemporary material been included

**SIGNIFICANCE**

This is a value judgment of the item, in the context of the relevant research area

• Is the item meaningful? (this incorporates feasibility, utility and relevance)

• Does it add context?

• Does it enrich or add something unique to the research?

• Does it strengthen or refute a current position?

• Would the research area be lesser without it?

• Is it integral, representative, typical?

• Does it have impact? (in the sense of influencing the work or behaviour of others)

**Quality assessment of academic papers**

| **First author, date** | **1** | **2** | **3** | **4** | **5** | **6** | **7** | **8** | **9** | **10** |
| --- | --- | --- | --- | --- | --- | --- | --- | --- | --- | --- |
| Akobo and Stewart, 2020 | Not clear | yes | yes | Not clear | yes | no | Not clear | yes | yes | yes |
| Avendano and Panico, 2018 | yes | yes | yes | yes | Partly – family health is mentioned but the outcome measures do not cover many dimensions of family health and the study used employers’ offer of flexible work, rather than parents’ use of flexible work | yes | yes | Yes – though other outcomes relating to wellbeing could have been used | yes | yes |
| Chandola et al 2019 | yes | yes | yes | yes | yes | yes | yes | yes | yes | yes |
| Charman and Tyson, 2024 | yes | yes | yes | Yes – though with limits | yes | no | yes | yes | yes | yes |
| Chung, 2018 | yes | yes | yes | yes | yes | yes | yes | yes | yes | unclear |
| Chung, 2020 | yes | yes | yes | yes | yes | yes | yes | yes | yes | unclear |
| Chung and Seo, 2024 | yes | yes | yes | yes | yes | yes | yes | yes | yes | unclear |
| Chung and van der Horst, 2020 | yes | yes | yes | yes | yes | yes | yes | yes | yes | unclear |
| Chung, 2019 | yes | yes | yes | yes | yes | yes | yes | yes | yes | yes |
| Griffiths et al., 2022 | yes | yes | yes | yes | yes | Yes – in part issue relating to COVID-19 | yes | yes | yes | yes |
| Griffiths et al., 2022 | yes | yes | yes | yes | yes | yes | yes | yes | yes | yes |
| Hobson and Dennis, 2024 | yes | yes | yes | yes | yes | yes | yes | yes | yes | yes |
| Hoque and Bacon, 2022 | yes | yes | yes | yes | yes | yes | yes | yes | yes | yes |
| Javed, 2019 | yes | yes | yes | yes | yes | yes | yes | yes | yes | yes |
| Kley and Reimer, 2023 | yes | yes | yes | yes | yes | yes | yes | yes | yes | yes |
| Nielsen and Yarker, 2023 | yes | yes | yes | yes | yes | yes | yes | yes | yes | yes |
| Petty et al., 2022 | yes | yes | yes | Yes –particularly focused on selected organisations, committed to improving their employees’ wellbeing through recognised schemes | yes | yes | yes | yes | yes | yes |
| Wang and Cheng, 2024 | yes | yes | yes | yes | yes | yes | yes | yes | yes | yes |
| Wang et al, 2022 | yes | yes | yes | yes | yes | yes | yes | yes | yes | yes |
| Wheatley, 2017 | yes | yes | yes | yes | yes | yes | yes | Unsure – at times difficult to follow | Unsure - difficult to follow at times | yes |
| Wheatley et al., 2023 | yes | yes | yes | yes | yes | yes | yes | yes | yes | yes |

1. Did the study address a clearly focused issue - was there a clear statement of the aims of the research?
2. Did the authors use an appropriate method to answer their question?
3. Was the research design appropriate to address the aims of the research?
4. Was any recruitment appropriate to the aims of the research?
5. Was the data items measured/data collected in a way that addressed the research issue?
6. Does the study appropriately reflect on limitations or issues, as appropriate to the design of the research?
7. Was the data analysis sufficiently rigorous?
8. Are the results presented clearly?
9. Is there a clear statement of findings?
10. Is there a statement about how valuable the research is?

For each, Yes, No, Unclear
